# Exercise Stress Echocardiography Predicts Adverse Cardiovascular Events in Hypertrophic Cardiomyopathy: A 5-Year Prospective Study

**DOI:** 10.31083/RCM41532

**Published:** 2025-10-22

**Authors:** Ye Su, Chunmei Li, Qionghui Peng, Lixue Yin

**Affiliations:** ^1^Department of Cardiovascular Ultrasound & Noninvasive Cardiology, Sichuan Provincial People's Hospital, University of Electronic Science and Technology of China, 610031 Chengdu, Sichuan, China; ^2^Ultrasound Medicine and Computational Cardiology Key Laboratory of Sichuan Province, Sichuan Provincial People's Hospital, University of Electronic Science and Technology of China, 610031 Chengdu, Sichuan, China

**Keywords:** exercise stress echocardiography, hypertrophic cardiomyopathy, risk stratification

## Abstract

**Background:**

Hypertrophic cardiomyopathy (HCM) is an autosomal dominant genetic disorder and a primary cause of sudden cardiac death (SCD) in young individuals. Studies have demonstrated that “left atrial strain” serves as a predictive marker for adverse cardiovascular events in diseases such as heart failure with preserved ejection fraction, moderate aortic stenosis, and diastolic dysfunction. Therefore, this study used exercise stress echocardiography (ESE) to identify high-risk factors in the early stages of HCM.

**Methods:**

A total of 142 HCM patients, diagnosed at the Sichuan Provincial People's Hospital in Chengdu, China, between 2017 and 2018, were included, along with 80 age- and gender-matched normal controls. ESE was employed to examine all subjects, and a 5-year follow-up of the HCM patients was conducted. HCM patients were classified into positive event and non-event groups based on follow-up results. Comparisons were made between the groups, focusing on left atrial reservoir strain, conduit strain, contractile strain, left ventricular global longitudinal strain at rest and peak exercise, and strain reserve.

**Results:**

(1) Significant impairments in global longitudinal strain (GLS), left atrial reservoir strain (LASr), and reserve function were observed in the positive events group: the resting (R) 4D and 2D GLS (R_4D_GLS: –13.20 ± 3.35; R_2D_GLS: –17.13 ± 3.71), and peak (P) 2D GLS (P_2D_GLS: –14.45 ± 3.51) were reduced (*p* < 0.05), accompanied by deteriorated GLS reserves (Δ2D_GLS: –2.68 ± 2.78; Δ2D_GLS %: –13.57% ± 18.89%;* p* < 0.05). The resting 2D and 4D left atrial (LA) reservoir strain at end-diastole (R_LASr_ED: 14.36 ± 5.52; R_4D_LASr: 10.30 ± 3.24) and peak 2D LASr (P_LASr_ED: 12.18 ± 5.71) were significantly impaired (*p* < 0.05), with a notable loss in reserve capacity (ΔLASr_ED: –2.18 ± 4.03; ΔLASr_ED %: –14.19% ± 27.85%; *p* < 0.05). (2) Correlations: positive events demonstrated strong correlations with R_4D_LASr (*r* = –0.67), R_LASr_ED (*r* = –0.58), P_LASr_ED (*r* = –0.61), and P_2D_GLS (*r* = 0.58). The positive events showed a weak linear association with the rest left ventricular outflow tract pressure gradient (R_LVOT-PG)(r = 0.35) and an “inverted U-shaped” relationship with the peak left ventricular outflow tract pressure gradient (P_LVOT-PG). (3) Logistic regression and collinearity analysis showed that the R_4D_LASr (odds ratio (OR) = 0.655, 95% confidence interval (CI) 0.547–0.783) and P_2D_GLS (OR = 1.383, 95% CI 1.142–1.675) were independent predictors for positive events.

**Conclusions:**

ESE provides critical information to predict risk factors in HCM patients: R_4D_LASr and P_2D_GLS have independent predictive values for positive cardiovascular events, which can assist in clinical assessment and the identification of high-risk HCM patients, promote individualized and precise risk stratification of HCM in clinical practice, and improve long-term prognosis.

## 1. Introduction

The latest Guidelines for the Diagnosis and Treatment of Hypertrophic Cardiomyopathy in Chinese Adults 2023 [1] indicate that hypertrophic cardiomyopathy (HCM) is a disease primarily characterized by myocardial hypertrophy caused by pathogenic mutations in genes encoding sarcomeric and/or related proteins. It remains one of the leading causes of sudden cardiac death (SCD) in young individuals. The incidence of HCM in the general population has gradually increased from 1 in 500 to 1 in 200 in recent years, showing a persistent upward trend [2, 3]. The 2014 European Society of Cardiology (ESC) Guidelines [4] and the 2020 American Heart Association/American College of Cardiology (AHA/ACC) Guidelines [5] explicitly state that stress echocardiography can evaluate and provide abundant prognostic information (COR IIa), which can serve as a reference for the clinical treatment and follow-up in HCM patients. The SCD risk assessment model for HCM, recommended by the 2020 AHA/ACC [5], includes seven factors: “maximum wall thickness, ventricular tachycardia, syncope, family history of SCD, ventricular aneurysm, left ventricular ejection fraction (LVEF), and late gadolinium enhancement on cardiac magnetic resonance imaging”, all of which involve at least three different types of examinations. However, some HCM patients are unable to complete these examinations due to economic conditions or contraindications. Currently, “left atrial strain” has been verified in several studies [6, 7, 8, 9, 10] to have predictive value for adverse cardiovascular events in diseases such as heart failure with preserved ejection fraction, chronic kidney disease, moderate aortic valve stenosis, and diastolic dysfunction. Therefore, in this study, we hypothesized that “left atrial reservoir strain” has clinical significance in predicting adverse cardiovascular events in HCM patients. The aim of this study was to identify and assess methods and parameters in high-risk HCM patients using exercise stress echocardiography (ESE) that could be easily and reliably used in everyday clinical practice.

## 2. Materials and Methods

### 2.1 Research Subjects 

HCM patients, clinically diagnosed at the Sichuan Provincial People’s Hospital in Chengdu, China, between 2017 and 2018, were included. The diagnostic criteria for HCM were based on the Guidelines for the Diagnosis and Management of HCM released by the 2014 ESC [4], which define HCM as the presence of wall thickness of one or more left ventricular myocardial segments ≥15 mm by any imaging modality, or ≥13 mm with a positive family history or positive genetic testing, not attributable to load factors. Exclusion criteria were as follows: patients with coronary heart disease, as shown by coronary computed tomography (CT) or coronary angiography; hypertension; moderate or severe aortic stenosis; congestive heart failure; patients with persistent or permanent atrial fibrillation or atrial flutter, and other diseases that could cause myocardial hypertrophy, as well as patients with poor imaging quality or those unable to cooperate with a 5-year follow-up. The control group consisted of individuals with no clinical cardiovascular history, normal results from treadmill exercise stress echocardiography, coronary angiography, or coronary CT, and who were matched for age and gender with the HCM group.

**Fig. 1.**
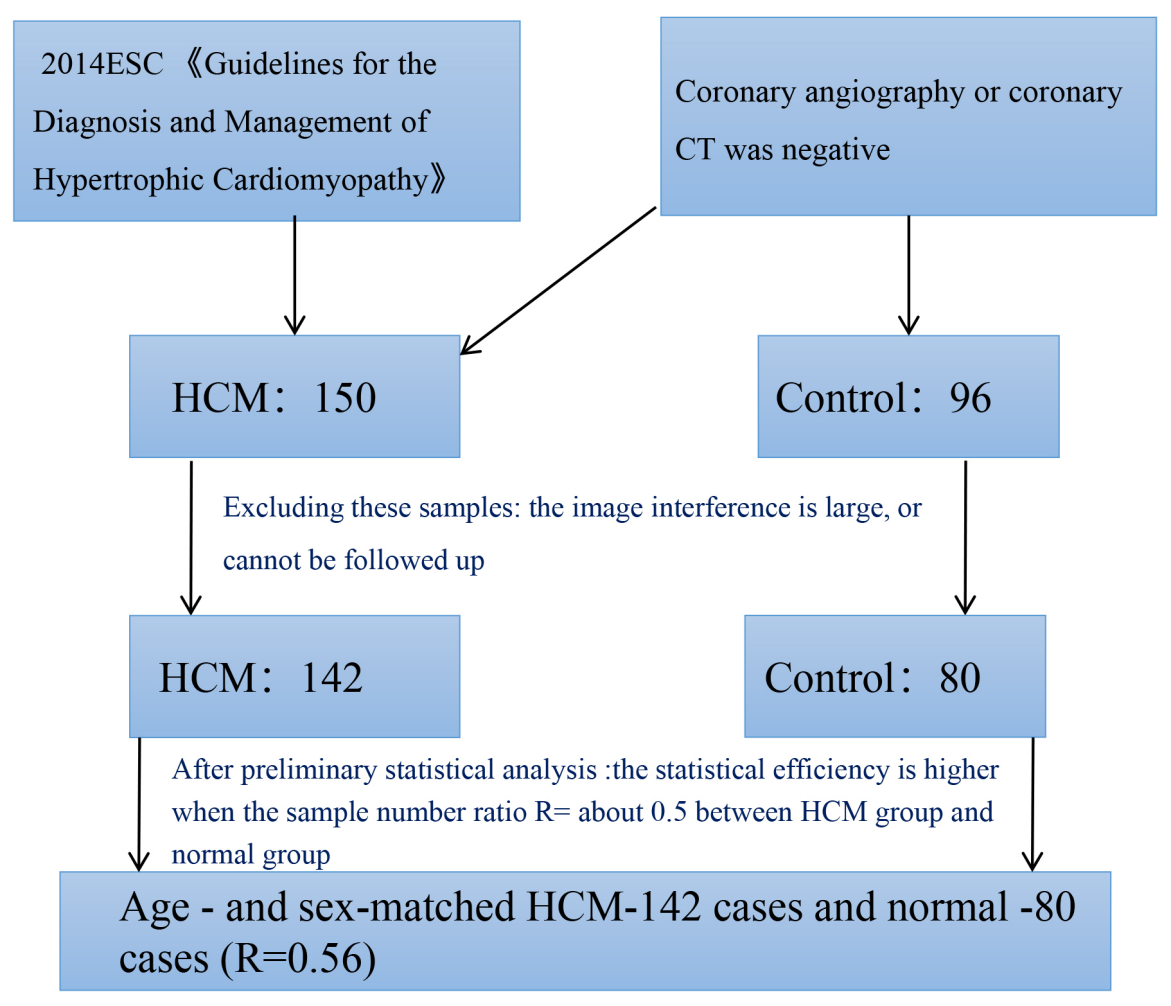
**Sample size of HCM and normal control group**. ESC, European Society of Cardiology; HCM, hypertrophic cardiomyopathy; CT, computed tomography.

The final sample size included 142 patients in the HCM group and 80 normal controls in the control group, as shown in Fig. 1. This study was approved by the Ethics Committee of the Sichuan Provincial People’s Hospital, and informed consent was obtained from all subjects.

### 2.2 Procedure

The HCM patients included in the study were those who were first diagnosed in our hospital and had not taken any drugs related to cardiovascular diseases before the test. Both the HCM group and the control group underwent ESE in sequential steps: resting echocardiography→ treadmill exercise→ peak stress echocardiography.

Symptom-restricted electrocardiography of treadmill exercise was performed by SunTechTango synchronized ambulatory hemometry (SunTech Medical Instruments, NC, USA) and a MortaraXScribe treadmill exercise analysis system (Mortara Instrument, Milwaukee, WI, USA) using the BRUCE protocol [11]. Electrocardiograms (ECGs) and blood pressure were monitored during exercise. All subjects were asked to stop β-blockers or calcium channel blockers for at least 24 hours before the trial. Resting contraction and diastolic blood pressure were measured, and ECGs were recorded simultaneously. As per the ACC/AHA 2002 Exercise Testing Guidelines [11], when the subjects reached the exercise termination criteria (target heart rate or the appearance of symptoms), the exercise was immediately terminated.

For rest and peak stress Echocardiography, we used GE-Vivid E95 color Doppler ultrasonic diagnostic instrument (E95, GE Medical Systems, Milwaukee, WI, USA), and 4V-D full volume probe (frequency 1.5–4.0 MHz, GE Medical Systems, Milwaukee, WI, USA), Philips-EPIQ7C ultrasonic diagnostic instrument (EPIQ7C, Philips Healthcare, Netherlands), X5-1 fully functional pure wave single crystal matrix probe (frequency 1.0–5.0 MHz, Philips Healthcare, Netherlands), QLAB quantitative analysis software (13.0, Philips Healthcare, Netherlands) and ECHO-PAC analysis software (203, GE Medical Systems, Milwaukee, WI, USA). Apical four-chamber, three-chamber, and two-chamber dynamic images from at least five cycles at rest and peak stage were collected.

### 2.3 Follow-up and Grouping

Subjects were monitored monthly via telephone for a continuous period of 5 years. The clinical endpoint was defined as the first occurrence of any adverse event, including heart failure, ventricular tachycardia, atrial fibrillation, defibrillator implantation, or unexplained syncope. Based on the outcomes, HCM patients were classified into the positive events group and the non-events group.

### 2.4 Image Data Analysis

**Fig. 2.**
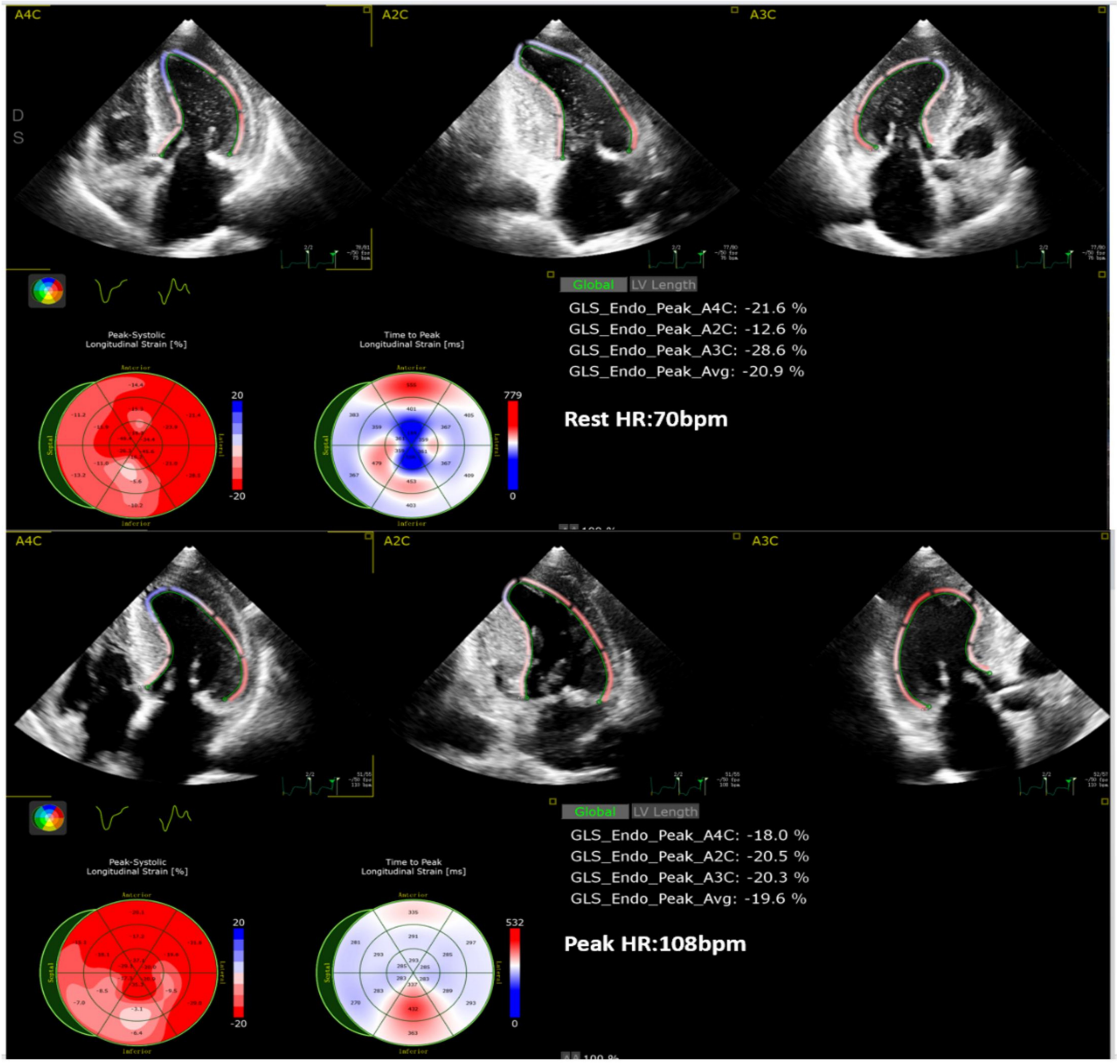
**Analysis of R_2D_GLS and P_2D_GLS (2D-STI)**. GLS, global longitudinal strain; HR, heart rate; 2D-STI, two-dimensional speckle-tracking imaging.

All images were measured and analyzed following the guidelines of the American Society of Echocardiography (ASE) [12]. Left atrial (LA) analysis was standardized according to the consensus on strain imaging established by the EACVI/ASE/Industry Task Force [13]. LA strain included LA reservoir strain, LA conduit strain, and LA contraction strain. When using the R-R gating analysis, the reservoir strain value (LASr) is positive, while the conduit strain (LAScd) and contraction strain (LASct) values are negative. The following parameters were included:

① In 2D mode, the mechanical parameters of the left ventricle (LV) at rest and peak were analyzed using two-dimensional speckle-tracking imaging (2D-STI) from the resting and peak A4C, A2C, and A3C views of the LV. Comprehensive calculations were performed to obtain the resting and peak global longitudinal strain (GLS) values (R_2D_GLS and P_2D_GLS) as shown in Fig. 2. The LVEF was measured using the 2D Simpson method at rest and peak.

**Fig. 3.**
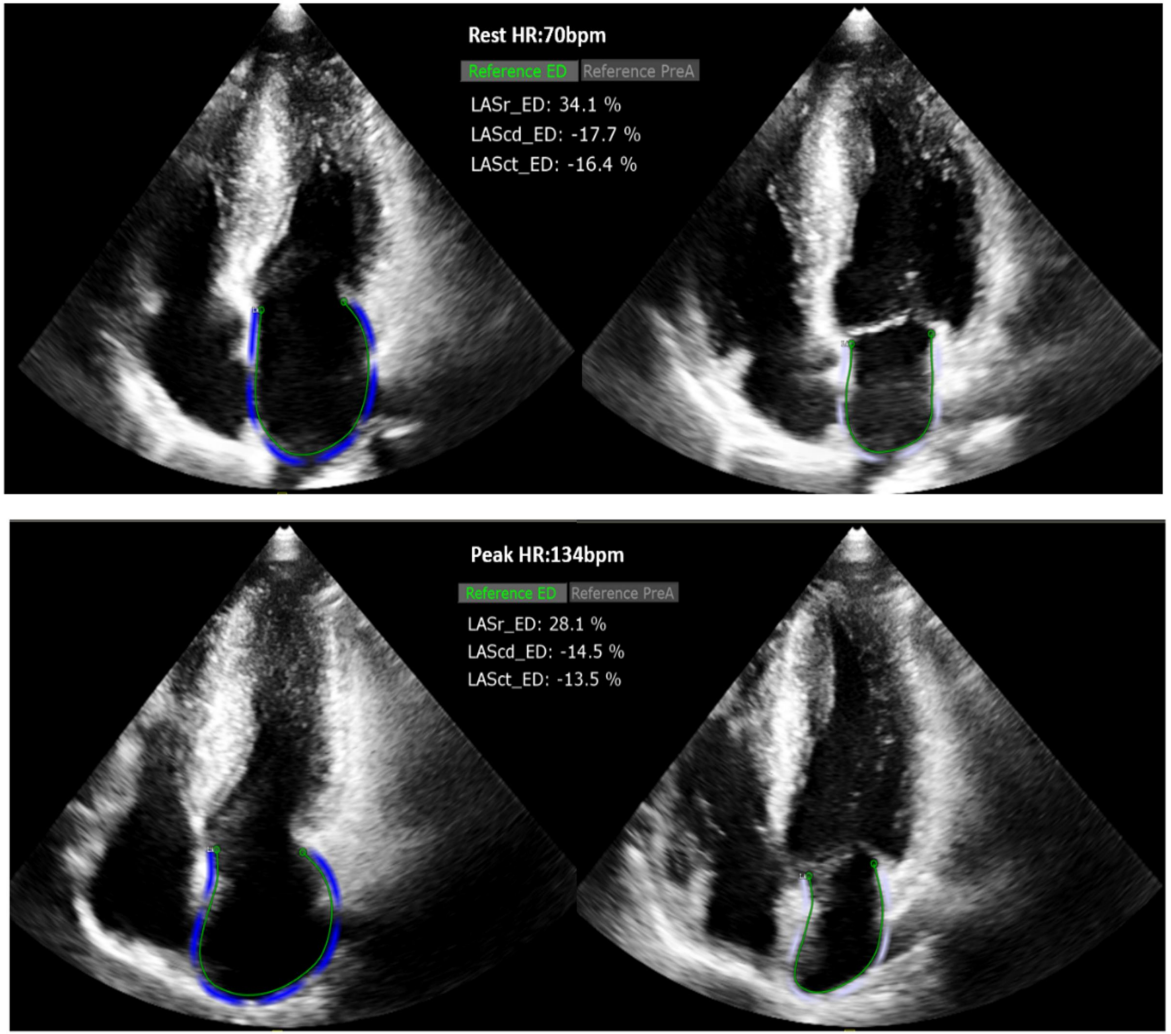
**Analysis of R_LASr_ED and P_LASr_ED (2D-STI)**. HR, heart rate; LASr, left atrial reservoir strain; LAScd, left atrial conduit strain; LASct, left atrial contraction strain; ED, end-diastole.

② In 2D mode, the mechanical parameters of the LA at rest and peak were analyzed using 2D-STI from the resting and peak A4C views of the LA. Comprehensive calculations were performed to obtain the resting and peak 2D left atrial reservoir strain (R_LASr_ED and P_LASr_ED), conduit strain (R_LAScd_ED and P_LAScd_ED), and contraction strain (R_LASct_ED and P_LASct_ED), as shown in Fig. 3.

③ In 2D mode, the structural, hemodynamic, and Doppler parameters of the LV at rest and peak were assessed, including maximum wall thickness at rest (R_MWT), measured at the LV short-axis at the mitral valve, papillary muscle, and apical levels in 2D. The early diastolic mitral inflow velocity at rest and peak (R_E and P_E), the average velocity of the septal and lateral mitral annulus during early diastole at rest and peak (R_e’ and P_e’), and the E/e’ ratio were measured in the apical A4C view. The resting left ventricular outflow tract pressure gradient (LVOT-PG) and peak LVOT-PG (R_LVOT-PG and P_LVOT-PG) and LA diameter (R_LAd) were also measured.

④ In 2D mode, the mechanical reserve parameters of LV: Absolute reserve of GLS: Δ2D_GLS = P_2D_GLS –R_2D_GLS, Relative reserve: Δ2D_GLS % = (Δ2D_GLS/R_2D_GLS) × 100%.

⑤ In 2D mode, the mechanical reserve parameters of LA: Absolute reserve of reservoir strain: ΔLASr = P_LASr –Rest_LASr, relative reserve of reservoir strain: ΔLASr % = (ΔLASr/Rest_LASr) × 100%, absolute reserve of conduit strain: ΔLAScd = Peak_LAScd – Rest_LAScd, relative reserve of conduit strain: ΔLAScd % = (ΔLAScd/Rest_LAScd) × 100%, absolute reserve of LA systolic strain: ΔLASct = Peak_LASct – Rest_LASct, relative reserve of systolic strain: ΔLASct % = (ΔLASct/Rest_LASct) × 100%.

**Fig. 4.**
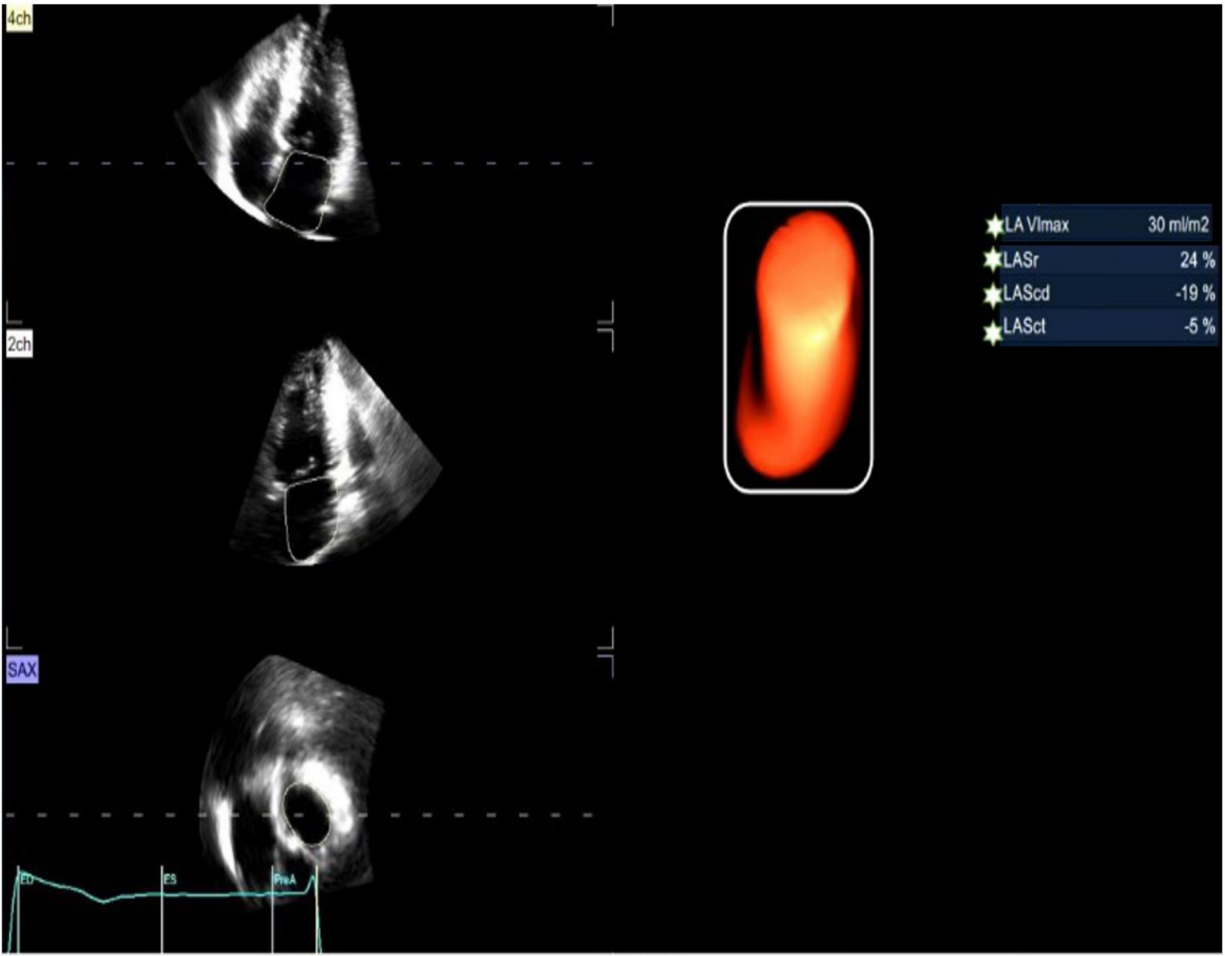
**Analysis of LA_Vlmax, R_4D_LASr, R_4D_LAScd, R_4D_LASct (3D-STI)**. LA_Vlmax, left atrium maximum volume index; LASr, left atrial reservoir strain; LAScd, left atrial conduit strain; LASct, left atrial contraction strain; 3D-STI, three-dimensional speckle-tracking imaging.

⑥ In the 3D mode, the mechanical parameters of the resting LA: 3D-STI was used to analyze the resting full-volume dynamic images of the left atrium. Through comprehensive calculation, the resting 4D left atrial reservoir strain (R_4D_LASr), resting 4D left atrial conduit strain (R_4D_LAScd), resting 4D left atrial contraction strain (R_4D_LASct), and the maximum volume index of the left atrium at rest (R_LA Vlmax) were obtained, as shown in Fig. 4.

⑦ In the 3D mode, the structural parameters of the resting LV: By analyzing the rest full-volume dynamic images of the apical four-chamber (A4C) view, the 4D rest global longitudinal strain (R_4D_GLS) can be obtained, and the left ventricular mass index (LVMI) was obtained.

⑧ Exercise parameters: heart rate (HR), metabolic equivalent (METS).

### 2.5 Image and Data Quality Control

Prior to the detection, patients with poor acoustic conditions or those unable to produce satisfactory images were excluded. Individualized breathing and short breath-holding training were provided to subjects based on the characteristics of their images, ensuring that dynamic images collected at rest and peak exercise were not obstructed by lung gas. This approach aimed to minimize or eliminate image interference and enhance image quality. A senior chief physician conducted a second review of all dynamic images, and Intra-class correlation (ICC) analysis was performed on the detection data to assess inter-observer consistency and ensure the reliability and repeatability of the results.

### 2.6 Statistical Methods 

Measurement data are presented as the mean ± standard deviation, and categorical data are expressed as percentages (%). Measurement data were compared using the independent samples *t*-test, and categorical data were compared using the chi-square test and Fisher’s exact test. Correlation matrix analysis, Collinearity analysis, Multivariate linear logistic regression and stratified analysis were used to identify parameters with predictive value for positive events. Receiver operating characteristic (ROC) and area under the curve (AUC) analyses were used to assess the sensitivity and specificity of each index in predicting positive events. *p* < 0.05 was considered statistically significant.

## 3. Results

### 3.1 Follow-up of Positive Events and Grouping

The clinical endpoint events were adjudicated as follows. Any of the following events which occurred for the first time in HCM patients during the 5-year follow-up period were regarded as clinical endpoints: heart failure, ventricular tachycardia, atrial fibrillation, defibrillator implantation, or unexplained syncope. There were no records of patients experiencing two or more events simultaneously, there were no deaths, no transplants, and no septal resections during the monthly telephone follow-up of all HCM patients in this study. As shown in Table 1, 40 positive cardiovascular events occurred in 40 HCM patients. The HCM patients were classified into the positive events group (40 cases) and the non-events group (102 cases).

**Table 1.** **Positive events in HCM**.

HCM group	Syncope	Heart failure	ICD or pacemaker implantation	Ventricular tachycardia	Atrial fibrillation
Positive events (N = 40)	6 cases	15 cases	6 cases	4 cases	9 cases
Non-events (N = 102)	0 case	0 case	0 case	0 case	0 case

HCM, hypertrophic cardiomyopathy; ICD, implantable cardioverter-defibrillator.

### 3.2 General Data and Cardiac Stress Function of HCM and Control Group

The HCM patients included in the study were those who were first diagnosed in our hospital and had not taken any drugs related to cardiovascular diseases before the test. In the HCM group, both R_2D_GLS and P_2D_GLS, as well as Δ2D_GLS and Δ2D_GLS %, were significantly reduced. Additionally, the R_LAd, the LVMI, the resting and peak LA reservoir strain, conduit strain, and contraction strain, along with ΔLASr, ΔLASr %, ΔLAScd, ΔLAScd %, ΔLASct and ΔLASct %, were all impaired, as shown in Table 2.

**Table 2.** **Clinical data and cardiac stress function between HCM group and control group**.

	HCM group	Control group	*p*
Sample	142	80	
Gender = 1 (%)	98 (69.00)	56 (70.00)	0.999
Age (mean (SD)) (year)	49.09 (14.17)	47.98 (10.61)	0.507
Smoke = 1 (%)	17 (11.97)	10 (12.50)	0.290
BSA (mean (SD)) (m^2^)	1.73 (0.20)	1.73 (0.20)	0.977
BMI (mean (SD)) (kg/m^2^)	24.10 (3.54)	24.03 (3.29)	0.888
METS (mean (SD))	9.03 (2.75)	10.57 (0.85)	<0.001*
LVMI (mean (SD)) (g/m^2^)	145.89 (60.56)	109.22 (26.13)	<0.001*
R_MWT (mean (SD)) (mm)	20.48 (4.75)	9.10 (1.12)	<0.001*
R_LAd (mean (SD)) (mm)	37.90 (5.30)	32.69 (2.79)	<0.001*
R_E/e’ (mean (SD))	13.58 (7.22)	8.11 (1.84)	<0.001*
R_LVOT-PG (mean (SD)) (mmHg)	17.79 (36.07)	4.84 (1.79)	<0.001*
R_LA_Vlmax (mean (SD)) (mm/m^2^)	34.94 (13.07)	20.84 (6.45)	<0.001*
R_HR (mean (SD)) (bpm)	77.56 (12.27)	88.17 (11.22)	<0.001*
R_LVEF (mean (SD)) (%)	71.92 (6.86)	66.43 (5.19)	<0.001*
P_HR (mean (SD)) (bpm)	155.40 (25.31)	159.18 (13.30)	0.147
P_LVOT-PG (mean (SD)) (mmHg)	35.68 (53.40)	9.42 (2.25)	<0.001*
P_E/e’ (mean (SD))	14.98 (6.67)	7.16 (1.17)	<0.001*
P_LVEF (mean (SD)) (%)	83.59 (6.99)	74.39 (5.62)	<0.001*
ΔEF (mean (SD)) (%)	11.62 (6.16)	7.96 (2.43)	<0.001*
ΔEF % (mean (SD))	16.78 (9.61)	12.07 (3.90)	<0.001*
R_2D_GLS (mean (SD)) (%)	–19.50 (3.29)	–21.01 (2.52)	<0.001*
P_2D_GLS (mean (SD)) (%)	–18.64 (4.68)	–24.57 (3.93)	<0.001*
Δ2D_GLS (mean (SD)) (%)	–0.86 (3.41)	3.56 (2.32)	<0.001*
Δ2D_GLS % (mean (SD))	–4.13 (18.23)	16.77 (10.83)	<0.001*
R_LASr_ED (mean (SD)) (%)	20.60 (6.95)	34.72 (11.17)	<0.001*
R_LAScd_ED (mean (SD)) (%)	–12.48 (6.73)	–23.07 (7.43)	<0.001*
R_LASct_ED (mean (SD)) (%)	–8.10 (4.37)	–11.56 (5.38)	<0.001*
P_LASr_ED (mean (SD)) (%)	19.95 (8.13)	54.10 (13.98)	<0.001*
P_LAScd_ED (mean (SD)) (%)	–12.24 (7.52)	–31.83 (8.75)	<0.001*
P_LASct_ED (mean (SD)) (%)	–7.39 (5.39)	–22.27 (10.12)	<0.001*
ΔLASr_ED (mean (SD)) (%)	–0.65 (5.47)	19.38 (7.24)	<0.001*
ΔLASr_ED % (mean (SD))	–3.03% (2.68%)	61.33% (30.14%)	<0.001*
ΔLAScd_ED (mean (SD)) (%)	–0.23 (6.83)	8.76 (6.52)	<0.001*
ΔLAScd_ED % (mean (SD))	12.29% (6.82%)	44.06% (37.08%)	<0.001*
ΔLASct_ED (mean (SD)) (%)	–0.71 (4.76)	10.71 (8.22)	<0.001*
ΔLASct_ED % (mean (SD))	6.33% (10.43%)	110.86% (98.05%)	<0.001*

*p < 0.05. METS, metabolic equivalent; LVMI, left ventricular mass index; LA, left atrial; LVOT-PG, left ventricular outflow tract pressure gradient; HR, heart rate; GLS, global longitudinal strain; LASr, left atrial reservoir strain; LAScd, left atrial conduit strain; LASct, left atrial contraction strain; LVEF, left ventricular ejection fraction; BSA, body surface area; BMI, body mass index; MWT, maximum wall thickness; LAd, left atrial diameter.

### 3.3 Cardiac Stress Function Between Positive Events Group and Non-Events Group

As shown in Table 3, in the positive events group, the R_4D_GLS, R_2D_GLS, and P_2D_GLS values were significantly worse, and Δ2D_GLS and Δ2D_GLS % were in a deteriorated state. The R_LASr_ED, R_LAScd_ED, R_4D_LASr, R_4D_LAScd, and R_4D_LASct values were significantly worse, and ΔLASr_ED and ΔLASr_ED % showed marked deterioration or even complete loss.

**Table 3.** **Clinical data and cardiac stress function between positive events group and non-events group**.

	Non-events group	Positive events group	*p*
Sample	102	40	
Gender = 1 (%)	73 (71.6)	25 (62.5)	0.396
Age (mean (SD)) (year)	46.32 (13.02)	56.15 (14.68)	<0.001*
Smoke = 1 (%)	21 (20.58)	7 (17.50)	0.977
BSA (mean (SD)) (m^2^)	1.76 (0.20)	1.66 (0.20)	0.008*
BMI (mean (SD)) (kg/m^2^)	23.94 (3.52)	24.49 (3.49)	0.403
Obstruction occurs = 1 (%)	21 (20.6)	18 (45.0)	0.006*
R_MWT (mean (SD)) (mm)	19.74 (4.50)	22.38 (4.88)	0.003*
R_LAd (mean (SD)) (mm)	36.64 (4.92)	41.13 (4.89)	<0.001*
R_E/e’ (mean (SD))	12.50 (5.24)	16.35 (10.32)	0.029*
R_LVOT-PG (mean (SD)) (mmHg)	9.97 (20.11)	37.74 (55.59)	0.004*
R_LA_Vlmax (mean (SD)) (mm/m^2^)	31.47 (8.50)	43.80 (17.86)	<0.001*
R_HR (mean (SD)) (bpm)	77.04 (11.21)	78.90 (14.70)	0.418
R_LVEF (mean (SD)) (%)	72.44 (6.43)	70.78 (7.80)	0.194
P_HR (mean (SD)) (bpm)	161.44 (22.45)	140.00 (25.94)	<0.001*
P_E/e’ (mean (SD))	13.65 (5.62)	18.39 (7.90)	0.001*
P_LVOT-PG (mean (SD)) (mmHg)	28.59 (44.11)	53.78 (69.33)	0.038*
P_LVEF (mean (SD)) (%)	84.86 (5.23)	80.35 (9.56)	0.007*
ΔEF (mean (SD)) (%)	12.42 (5.66)	9.58 (6.94)	0.013*
ΔEF % (mean (SD))	30.62% (2.86%)	31.52% (4.12%)	0.140
R_2D_GLS (mean (SD)) (%)	–20.43 (2.59)	–17.13 (3.71)	<0.001*
R_4D_GLS (mean (SD)) (%)	–16.60 (3.22)	–13.20 (3.35)	<0.001*
R_LASr_ED (mean (SD)) (%)	23.05 (5.85)	14.36 (5.52)	<0.001*
R_LAScd_ED (mean (SD)) (%)	–14.29 (6.72)	–7.86 (4.05)	<0.001*
R_LASct_ED (mean (SD)) (%)	–8.53 (4.51)	–7.00 (3.81)	0.059
R_4D_LASr (mean (SD)) (%)	18.79 (5.01)	10.30 (3.24)	<0.001*
R_4D_LAScd (mean (SD)) (%)	–11.41 (5.09)	–5.13 (2.69)	<0.001*
R_4D_LASct (mean (SD)) (%)	–6.95 (4.51)	–5.18 (2.78)	0.022*
P_2D_GLS (mean (SD)) (%)	–20.28 (4.01)	–14.45 (3.51)	<0.001*
Δ2D_GLS (mean (SD)) (%)	–0.14 (3.38)	–2.68 (2.78)	<0.001*
Δ2D_GLS % (mean (SD))	–0.43% (16.64%)	–13.57% (18.89%)	<0.001*
P_LASr_ED (mean (SD)) (%)	22.99 (6.81)	12.18 (5.71)	<0.001*
P_LAScd_ED (mean (SD)) (%)	–14.09 (7.59)	–7.54 (4.85)	<0.001*
P_LASct_ED (mean (SD)) (%)	–8.46 (5.50)	–4.67 (3.99)	<0.001*
ΔLASr_ED (mean (SD)) (%)	–0.06 (5.85)	–2.18 (4.03)	0.015*
ΔLASr_ED % (mean (SD))	1.29% (2.52%)	–14.19% (2.79%)	0.002*
ΔLAScd_ED (mean (SD)) (%)	–0.20 (7.45)	–0.32 (4.95)	0.921
ΔLAScd_ED % (mean (SD))	8.18% (5.66%)	22.78% (9.14%)	0.351
ΔLASct_ED (mean (SD)) (%)	–0.07 (4.81)	–2.33 (4.28)	0.011*
ΔLASct_ED % (mean (SD))	13.28% (10.12%)	–11.38% (11.10%)	0.206

*p < 0.05. LA, left atrial; LVOT-PG, left ventricular outflow tract pressure gradient; HR, heart rate; GLS, global longitudinal strain; LASr, left atrial reservoir strain; LAScd, left atrial conduit strain; LASct, left atrial contraction strain; LVEF, left ventricular ejection fraction; BSA, body surface area; BMI, body mass index; MWT, maximum wall thickness; LAd, left atrial diameter.

### 3.4 Cardiac Stress Function Between Obstruction Group and Non-Obstruction Group 

As shown in Table 4, in the obstruction group, the R_2D_GLS, P_2D_GLS, R_4D_LASr, P_LASr_ED and P_LASct_ED values were significantly worse, both the Δ2D_GLS, Δ2D_GLS % and ΔLASr_ED, ΔLASr_ED %, ΔLASct_ED, ΔLASct_ED %, were in a deteriorated state.

**Table 4.** **Clinical data and cardiac stress function between obstruction group and non-obstruction group**.

	Non-obstruction group	Obstruction group	*p*
Sample	103	39	
Gender = 1 (%)	76 (73.8)	22 (56.4)	0.073
Age (mean (SD)) (year)	48.80 (14.12)	49.87 (14.44)	0.688
Positive = 1 (%)	22 (21.4)	18 (46.2)	0.006*
Mitral valve regurgitation = 1 (%)	21 (20.39)	8 (20.51)	0.862
Tricuspid regurgitation = 1 (%)	77 (74.75)	30 (76.92)	0.857
BSA (mean (SD)) (m^2^)	1.74 (0.19)	1.72 (0.25)	0.631
BMI (mean (SD)) (kg/m^2^)	24.11 (3.29)	24.07 (4.08)	0.949
LVMI (mean (SD)) (g/m^2^)	150.60 (64.24)	133.46 (48.10)	0.133
R_MWT (mean (SD)) (mm)	19.55 (4.20)	22.92 (5.27)	<0.001*
R_LAd (mean (SD)) (mm)	37.51 (5.22)	39.05 (5.32)	0.112
R_E/e’ (mean (SD))	13.15 (7.11)	14.72 (7.49)	0.250
R_LVOT-PG (mean (SD)) (mmHg)	6.28 (2.82)	48.20 (59.16)	<0.001*
R_LA_Vlmax (mean (SD)) (mm/m^2^)	34.82 (12.11)	35.26 (15.50)	0.859
R_HR (mean (SD)) (bpm)	76.08 (11.48)	81.49 (13.53)	0.018*
P_HR (mean (SD)) (bpm)	156.65 (26.34)	152.10 (22.36)	0.341
P_LVOT-PG (mean (SD)) (mmHg)	13.79 (8.38)	93.50 (75.26)	<0.001*
P_E/e’ (mean (SD))	13.76 (6.09)	18.21 (7.13)	<0.001*
R_4D_GLS (mean (SD)) (%)	–15.96 (3.53)	–14.97 (3.65)	0.084
R_2D_GLS (mean (SD)) (%)	–19.92 (3.26)	–18.38 (3.14)	0.012*
R_LASr_ED (mean (SD)) (%)	21.21 (7.10)	18.98 (6.36)	0.087
R_LAScd_ED (mean (SD)) (%)	–12.87 (7.07)	–11.44 (5.68)	0.260
R_LASct_ED (mean (SD)) (%)	–8.23 (4.65)	–7.77 (3.55)	0.576
R_4D_LASr (mean (SD)) (%)	17.23 (6.03)	14.21 (5.23)	0.006*
R_4D_LAScd (mean (SD)) (%)	–9.99 (5.50)	–8.72 (4.88)	0.207
R_4D_LASct (mean (SD)) (%)	–6.80 (4.53)	–5.54 (2.85)	0.108
P_2D_GLS (mean (SD)) (%)	–19.57 (4.61)	–16.20 (3.94)	<0.001*
P_LASr_ED (mean (SD)) (%)	21.15 (8.12)	16.76 (7.33)	0.004*
P_LAScd_ED (mean (SD)) (%)	–12.66 (8.10)	–11.15 (5.65)	0.287
P_LASct_ED (mean (SD)) (%)	–8.07 (5.46)	–5.60 (4.83)	0.015*
Δ2D_GLS (mean (SD)) (%)	–0.35 (3.30)	–2.18 (3.37)	0.004*
Δ2D_GLS % (mean (SD))	–1.52% (17.46%)	–11.01% (18.63%)	0.005*
ΔLASr_ED (mean (SD)) (%)	–0.06 (5.50)	–2.22 (5.12)	0.036*
ΔLASr_ED % (mean (SD))	0.20% (25.56%)	–11.79% (28.49%)	0.017*
ΔLAScd_ED (mean (SD)) (%)	–0.21 (7.42)	–0.29 (4.99)	0.951
ΔLAScd_ED % (mean (SD))	12.19% (66.62%)	12.52% (73.12%)	0.979
ΔLASct_ED (mean (SD)) (%)	–0.16 (4.67)	–2.16 (4.74)	0.025*
ΔLASct_ED % (mean (SD))	17.41% (114.65%)	–22.95% (1.93%)	0.039*

*p < 0.05. LVMI, left ventricular mass index; LA, left atrial; LVOT-PG, left ventricular outflow tract pressure gradient; HR, heart rate; GLS, global longitudinal strain; LASr, left atrial reservoir strain; LAScd, left atrial conduit strain; LASct, left atrial contraction strain; LVEF, left ventricular ejection fraction; BSA, body surface area; BMI, body mass index; MWT, maximum wall thickness; LAd, left atrial diameter.

### 3.5 Correlation and Logistic Regression and Collinearity Analysis

Positive events were negatively correlated with R_4D_LASr (r = –0.67), P_LASr_ED (r = –0.61), R_LASr_ED (r = –0.58), and positively correlated with P_2D_GLS (r = 0.58), R_4D_LAScd (r = 0.57), R_4D_GLS (r = –0.43), R_2D_GLS (r = 0.44), R_4D_LAScd (r = 0.57), P_LAScd_ED (r = 0.49), R_LA_Vlmax (r = 0.43) and R_LAScd_ED (r = 0.47). Positive events were weakly correlated with R_LVOT-PG (r = 0.35), and P_LVOT-PG had an “inverted U” shape relationship with positive events.

As shown in Table 5, the collinearity analysis revealed that the variance inflation factor (VIF) values of R_4D_LASr, P_LASr_ED, R_LA_Vlmax, P_2D_GLS and R_4D_GLS were less than 3, and the tolerance (TOL) were greater than 0.2, which indicates mild collinearity and is acceptable.

**Table 5.** **Collinearity analysis for HCM**.

	TOL	VIF	*p*
R_4D_LASr	0.53	1.91	<0.001
P_LASr_ED	0.54	1.85	0.005
R_LA_Vlmax	0.69	1.44	0.166
R_4D_GLS	0.68	1.46	0.243
P_2D_GLS	0.54	1.84	0.029
R_LVOT-PG	0.40	2.53	0.139
P_LVOT-PG	0.43	2.33	0.505

TOL, tolerance; VIF, variance inflation factor; LA, left atrial; LASr, left atrial reservoir strain; LA_Vlmax, left atrium maximum volume index; GLS, global longitudinal strain; LVOT-PG, left ventricular outflow tract pressure gradient.

As shown in Table 6, the logistic regression and stratified analysis revealed that the P of R_4D_LASr and P_2D_GLS were less than 0.05, which showed that R_4D_LASr and P_2D_GLS had independent predictive value for positive cardiovascular events, and the value of “Nagelkerke R^2^” = 0.720, the *p* value of “Hosmer and Lemeshow test” = 0.407, which indicated that the model has good explanatory power.

**Table 6.** **Logistic regression for HCM**.

	B	Standard error	*p*	Exp(B)	95% CI (Lower–Upper)
R_4D_LASr	–0.424	0.09	<0.001*	0.655	0.547–0.783
P_2D_GLS	0.324	0.09	0.001	1.383	1.142–1.675

*p < 0.05. GLS, global longitudinal strain; LASr, left atrial reservoir strain; HCM, hypertrophic cardiomyopathy.

### 3.6 The Predictive Efficiency for Positive Events

**Fig. 5.**
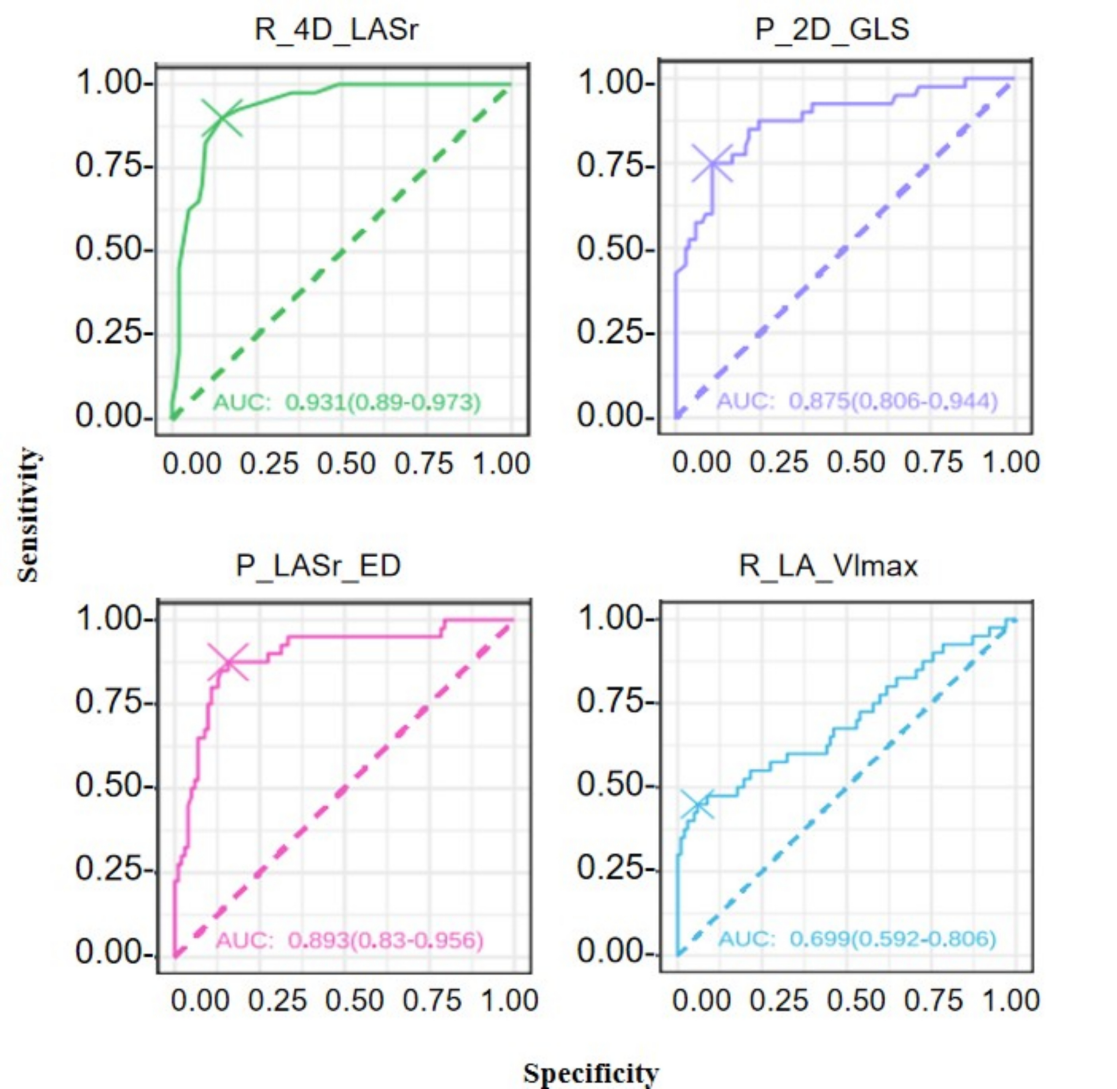
**Predictive power for positive cardiovascular events**. LA, left atrial; LASr, left atrial reservoir strain; GLS, global longitudinal strain; LA_Vlmax, left atrium maximum volume index.

Combining the number of patients with positive events and the principle of statistical efficiency, as well as the clinical significance of the above parameters, suggested that the following parameters can be included in the ROC analysis, as shown in Table 7 and Fig. 5, the prediction efficiency of R_4D_LASr, P_LASr_ED and P_2D_GLS is better than R_LA_Vlmax.

**Table 7.** **Predictive efficacy of cardiac function parameters of positive events**.

	Cutoff value	Specificity	Sensitivity	AUC
R_4D_LASr	14.50	85.29	90.00	0.93
P_LASr_ED	16.84	84.31	87.50	0.89
P_2D_GLS	–15.70	89.22	75.00	0.88
R_LA_Vlmax	44.62	59.00	45.00	0.70

AUC, area under the curve; LA, left atrial; LASr, left atrial reservoir strain; GLS, global longitudinal strain; LA_Vlmax, left atrium maximum volume index.

### 3.7 ICC Analysis

As shown in Table 8, the ICC analysis demonstrated good inter-observer consistency.

**Table 8.** **Inter-observer consistency analysis**.

	Inter-observe		
	ICC	95% Lower	95% Upper
R_4D_LASr	0.89	0.85	0.92
R_LASr_ED	0.91	0.88	0.94
P_2D_GLS	0.89	0.86	0.92
R_2D_GLS	0.89	0.86	0.92

ICC, intra-class correlation; GLS, global longitudinal strain; LASr, left atrial reservoir strain.

## 4. Discussion

In this study, ESE was used to conduct a comprehensive analysis of exercise-induced cardiac function and reserve in HCM patients, with a 5-year follow-up for positive cardiovascular events. We observed that in the HCM positive event group, both resting and peak left atrial reservoir strain and GLS were significantly impaired, accompanied by marked deterioration or even loss of reserve. R_4D_LASr and P_2D_GLS had independent predictive value for positive cardiovascular events.

Huang *et al*. [14] found that in HCM, whether the ventricular wall is thickened or not, there are abnormalities in GLS, and mechanical abnormalities may precede the occurrence of hypertrophy [15]. At the same time, through multivariate analysis, it was found that GLS reflects impaired myocardial contraction and is related to the severity of HCM [16]. Wu *et al*. [17] conducted further research on HCM and found that the GLS of HCM decreased both before and after exercise, the systolic reserve during exercise was also significantly reduced, and the decreased GLS at peak was significantly related to exercise intolerance. Further research by Badran *et al*. [18] found that the systolic function reserve of HCM patients decreased by 23% after exercise. Based on previous studies, this study combined the influencing factors of GLS. Not only did it select the most physiologically appropriate treadmill exercise stress mode for HCM patients, but according to the 5-year follow-up of HCM, it was found that even if the GLS of HCM patients at rest is at the edge of the critical value or slightly decreased, the GLS after exercise stress will immediately show a significant decrease, and the degree of reduction in systolic strain reserve is about 22%. In the positive events group, the GLS at rest and peak exercise is significantly decreased, and the reduction in systolic reserve (Δ2D_GLS %) is up to 33%, and the reserve capacity is in a deteriorated state. At the same time, we found that the peak GLS has predictive value for positive events. Because GLS is a parameter obtained by digital processing of sound images after detecting myocardial deformation by ultrasonic speckle-tracking imaging (STI), it reflects the quantitative and localized analysis of myocardial mechanics. The 2D-GLS obtained under 2D-STI is not affected by angle dependence and the motion interference of adjacent myocardial segments, so as to realize the continuous observation of myocardial motion, which has a better predictive value for the prognosis of cardiovascular diseases [19, 20] and has been regarded as one of the main tools for evaluating systolic function by the ASE/ESC [21]. However, 2D-GLS also has its own limitations and is easily affected by load factors. When the cardiac afterload significantly increases, its accuracy will be diminished. Therefore, in this study, the exercise stress mode was selected to make up for the shortcomings of speckle tracking technology, and a comprehensive evaluation was carried out on the resting and peak strains of HCM and the reserve capacity after load strain to reflect cardiac systolic function and reserve from a more objective perspective. The final result that “peak GLS has a more significant predictive efficacy for positive events” also supported this observation.

In the 2014 and 2022 ESC guidelines [4, 22] for the clinical risk factors for SCD in HCM, the atrial index is “left atrial internal diameter/size”, and the LA_Vlmax >34 mL/m^2^ is also considered a key parameter for determining heart failure with preserved ejection fraction. In this study, there was a statistically significant difference in the resting inner diameter of LA(R_LAd) between the HCM group and the control group. The resting left atrial volume index (R_LA_Vlmax) of HCM was significantly higher than that of the control group. However, usually clinical measurements are combined with the patient’s BSA. Therefore, LA_Vlmax should be more accurate, after all, the abnormal diastolic function of HCM leads to changes in the structure and function of the left atrium, the remodeling pattern in terms of structure is manifested as an increase in three-dimensional volume, which can be verified in the myocardial magnetic resonance 3D imaging study of HCM patients by Muresan *et al*. [23]. All the HCM patients enrolled in this study were capable of undergoing the treadmill exercise stress test, and were not in the subclinical heart failure or clinical heart failure stage. According to the occurrence and development of pathophysiological diseases and previous research results [7], the “left atrial size” is also affected by many factors such as the duration of the disease, the degree of disease development, and mitral regurgitation. Therefore, simply detecting “left atrial size” may not be sensitive and rigorous enough in terms of a pathological mechanism, which affects the accurate judgment of diastolic function and the risk of HCM in clinical practice, which is one of the reasons why the left atrial strain series parameters were introduced in this study.

Left atrial reservoir strain has been shown by previous studies to not only sensitively and objectively reflect diastolic dysfunction in HCM at an earlier stage, but also that the sensitivity and accuracy in predicting adverse cardiovascular events are significantly improved compared to resting GLS and E/e’ [24, 25, 26, 27, 28, 29, 30]. Left atrial storage strain has been proven to be closely related to pulmonary capillary wedge pressure and the early response to earlier treatment, and is a strong predictor of long-term prognosis in HCM patients [14, 31]. In this study, for the analysis of left atrial strain, regardless of 2D or 3D-STI, ventricular end-diastole was selected as the zero baseline of the left atrial strain curve, and longitudinal strain was generated from each atrial segment. It was found that the left atrial storage strain, conduit strain, and contraction strain in the positive events group were significantly worse than those in the non-events group. After exercise, the left atrial peak storage strain, conduit strain, and contraction strain in the positive events group were further reduced, and the reserve (ΔLASr_ED, ΔLASr_ED %) was significantly deteriorated. Both the regression analysis and the ROC found that “R_4D_LASr” had excellent predictive efficacy for positive events, since “R_4D_LASr” is combined with full volume real time dynamic images. It reflects the mechanical changes in both the longitudinal and circumferential directions, and it can reflect the mechanical changes during the diastolic process of the entire cardiac cycle without being limited by heart rate. It has less dependence on angles and loads, and it can sensitively distinguish the active and passive movements of myocardial tissue, thus objectively and comprehensively evaluating the storage, conduit, and contraction functions of the left atrium.

In all HCM patients, both resting obstruction and obstruction which occurred during exercise were labeled as obstruction patients. As shown in Table 4, in the non-event group (102 HCM patients), there were 21 cases of obstruction, accounting for 20.6%, and in the positive event group (40 HCM patients), there were 18 cases of obstruction, accounting for 45.0%, which was statistically significant (*p* = 0.006). In the obstruction group, the R_4D_GLS, R_2D_GLS, P_2D_GLS, R_LASr_ED, R_4D_LASr, P_LASr_ED and P_LASct_ED values were significantly worse, both the Δ2D_GLS, Δ2D_GLS % and ΔLASr_ED, ΔLASr_ED % were in a deteriorated state. Norrish *et al*. [32] also found an inverted U-shaped relationship between left ventricular hypertrophy and the risk of SCD. The influence of more factors on the risk of SCD needs to be further evaluated. In this study, we conducted correlation and logistic regression analyses on whether obstruction affects cardiovascular events and found that the positive events were weakly correlated with R_LVOT-PG (*r* = 0.35), and P_LVOT-PG had an “inverted U” shape relationship with positive events, which suggested that the probability of cardiovascular events does not simply increase with increasing outflow tract obstruction. Moreover, in the further multivariate progressive logistic regression analysis, it was also found that the predictive value of R_LVOT-PG and P_LVOT-PG for cardiovascular events in HCM is not independent, and does not play a decisive role independently. LVOT-PG can be affected by cardiac function. Maurizi *et al*. [33], in a multidisciplinary center study observed that even if outflow tract obstruction was successfully relieved, some patients would still progress to heart failure. Elliott *et al*. [34] found that the 5-year survival rate of all-cause mortality in patients with obstruction was significantly correlated with decreased cardiac function (New York Heart Association (NYHA) Class I: 91.0%; NYHA Class II: 83.3%; NYHA Class III/IV: 82.6%, *p* = 0.002), and that the sudden death mortality rate of asymptomatic left ventricular outflow tract obstruction (LVOTO) patients was relatively low. Maron *et al*. [35] found that although the overall mortality rate of patients with obstruction was significantly higher than that of patients without obstruction (relative risk, 2.0; *p* = 0.001), the likelihood of severe symptoms and death associated with obstruction did not increase with the increased threshold (≥30 mmHg) of the outflow gradient. Based on the fact that all the HCM patients enrolled in this study were able to complete the treadmill exercise stress ultrasound test, their exercise capacity and cardiac function were both at NYHA Class I, suggesting that the factor of “outflow tract obstruction” that we have been continuously concerned about for a long time may only be a manifestation. On the one hand, obstruction causes sharp and abnormal changes in short-term cardiac function and hemodynamics, as well as abnormal myocardial electrical activity. On the other hand, it triggers myocardial structure remodeling and reshaping of myocardial mechanical function and electrical activity, all of which may have a greater impact on cardiovascular events in HCM. This will require additional in-depth multimodal research at the molecular level to further elucidate these issues.

A retrospective study involving more than 3000 HCM patients showed that abnormal resting LV-GLS was related to adverse cardiovascular events [36]. In addition, a large study by Yang *et al*. [37] also confirmed that the impairment of resting GLS was significantly related to adverse cardiovascular events in HCM, and the GLS can assist in identifying and determining the risk degree of HCM, which also partially explains the high rate of SCD in young HCM patients. At the same time, left atrial strain has been proven to have predictive value for adverse cardiovascular events in clinical practice [16, 38]. The quantitative analysis of left atrial strain can also provide a basis for the diagnosis, classification, prediction of new atrial fibrillation, and identification of adverse cardiovascular events in subclinical left atrial dysfunction [39]. In this 5-year follow-up study, it was found that the GLS and its reserve, as well as the LASr and its reserve in the positive event group were worse than those in the non-event group. Logistic regression and collinearity analysis showed that R_4D_LASr and P_2D_GLS had independent predictive value for positive cardiovascular events. In Fig. 5 and Table 7, the R_4D_LASr and P_2D_GLS showed strong predictive ability for cardiovascular events in HCM. According to classical pathophysiology, abnormal diastolic function is not only an early pathophysiological feature of HCM but also one of the important reasons for the deterioration and progression of HCM. It strongly implies that positive events may be simultaneously influenced by both the systolic and diastolic functions of the ventricles and atria, with more emphasis on the diastolic function, and that these positive clinical events may be the result of a combination of multiple factors.

## 5. Limitations

Speckle tracking echocardiography suffers from a number of relevant technical limitations, such as the intervendor variability, dependency on optimal image quality, sufficient frame rates (typically ≥40 fps), operator expertise, loading conditions and chest wall conformation [40, 41, 42]. In future studies, we plan to expand the sample size, extend the follow-up duration, and incorporate a broader range of ultrasound systems with advanced strain analysis algorithms.

In the study, the endpoint events such as heart failure, ventricular tachycardia, atrial fibrillation, and syncope were direct manifestations of the natural progression or deterioration of the disease. Implantable cardioverter-defibrillator (ICD) implantation was a decision made by clinicians through comprehensive judgment, which indirectly reflects the deterioration of the disease to a certain extent. The main purpose of this study at this stage is to screen risk factors and prediction models. Therefore, “ICD implantation” is set as the “Primary Prevention”, which still inevitably leads to “bias” and “endpoint heterogeneity” at the current stage. In the subsequent stage, on the one hand, classical survival analysis or competitive risk analysis will be conducted, and on the other hand, more sensitive multimodal potential new markers will be added to continuously improve and enrich the results and conclusion.

At this stage of the study, no additional biological markers were included, such as B-type natriuretic peptide (BNP). This study considers that the pathophysiological mechanism of HCM shows that abnormal diastolic function is an early pathophysiological feature and also one of the important reasons for the deterioration and development of HCM. When designing the research protocol, the non-invasive method of stress ultrasound was given more priority. At the current stage, we hope to explore new non-invasive imaging parameters to predict and assess the risk of HCM. BNP is a “molecular marker” reflecting cardiac load and cardiac function. Its pathophysiological core is the response to increased ventricular pressure/volume load. Although it is widely used in the diagnosis, assessment and management of heart failure in clinical practice, it has certain limitations in the specific risk assessment of HCM: Elevated BNP is not only seen in HCM, but is also related to multiple factors such as age, renal insufficiency, atrial fibrillation, and hypertension. BNP has certain correlations with some parameters. After inclusion, it may reduce the model efficiency due to collinearity. At this stage of the study, all the enrolled patients were able to perform the treadmill exercise test, and the cardiac function was NYHA Class I. The clinical endpoint events were Syncope, Heart failure, ICD or pacemaker implantation, ventricular tachycardia, and atrial fibrillation. BNP is more likely to reflect events related to cardiac insufficiency (such as heart failure), has a relatively low predictive sensitivity for arrhythmia events such as SCD and atrial fibrillation, and has a weak correlation with the “composite cardiovascular events” of concern in this study. Although BNP was not included in this present study, its clinical value is not denied. Instead, it is a phased choice in the research design. Our aim is to integrate multi-omics and multimodal imaging technologies, further optimize the research protocol, and explore deeper mechanisms to improve the assessment of cardiac function and stratify the prognosis of HCM patients, ultimately enhancing their diagnosis, treatment and long-term management.

## 6. Conclusions

ESE provides critical information to predict risk factors in HCM patients: R_4D_LASr and P_2D_GLS had independent predictive value for positive cardiovascular events, which can assist in the clinical assessment, and identification of high-risk HCM patients, promote the individualized and precise risk stratification of HCM in clinical practice, and improve the long-term prognosis.

## Availability of Data and Materials

Since this study is still ongoing, the data are not currently fully publicly available. Upon completion of the subsequent research, the data can be made accessible upon reasonable request.
